# Supplementary material for: Are carbon emissions trading and green financial instruments synergistic? -Comprehensive quantitative research based on content analysis
Source: PLoS One. 2024 Mar 7;19(3):e0298601. doi: 10.1371/journal.pone.0298601 (PMC10919601; doi:10.1371/journal.pone.0298601)
Supplement: S1 Data — (ZIP) [file pone.0298601.s001.zip › Data/地级市Z_F工作报告.zip!/║╙▒▒/│╨╡┬╩╨/└┤╫╘╣1⁄2╓┌║┼í╛┬φ┐╦╩2╛▌═°í┐_2019.docx]

各位代表：

　　现在，我代表市人民政府向大会报告工作，请予审议，并请各位市政协委员和列席人员提出意见。

　　一、2018年工作回顾

　　刚刚过去的2018年，全市上下深入学习贯彻习近平新时代中国特色社会主义思想和党的十九大精神，全面落实党中央国务院、省委省政府重大决策部署，在市委坚强领导下，坚持稳中求进工作总基调，认真践行新发展理念，围绕“12345”总体思路目标，攻坚克难，奋发作为，较好完成了市十四届人大三次会议确定的各项目标任务，为建设新时代生态强市、魅力承德奠定了更加坚实基础。

　　（一）经济发展实现稳中有进。积极应对经济下行压力，全市经济保持平稳健康增长。预计全市生产总值增长6.4%；全部财政收入增长22%、首次跃上200亿元台阶，公共财政预算收入增长17.2%、突破100亿元，收入增速和质量均居全省前列；城乡居民人均可支配收入分别增长8.8%和10.8%，均高于全省平均水平。着力推进项目建设。实施重点项目“四个一”工程，在建亿元以上项目1006个、10亿元以上项目133个，竣工投产329个；29个省重点项目、220个市重点项目全面开工。着力扩大居民消费。社会消费品零售总额增长9.2%。深入推进“互联网+流通”行动，电子商务交易额实现808亿元、农产品上行规模达到12亿元，分别增长28%和21%。着力提升外资外贸水平。实际利用外资增长4倍，外贸出口4.9亿美元，新增出口备案企业30家，均超额完成年度目标任务。

　　（二）结构调整迈出坚实步伐。牢牢把握高质量发展根本要求，坚决去、主动调、加快转，积极培育新动能，全市绿色产业增加值占GDP比重达到39%，高新技术产业增加值增长16.3%，服务业对经济增长贡献率超过70%。大力培育绿色主导产业。德鸣大数据、华明服务外包产业园建设取得实质性进展。承钢120万吨高强冷轧板项目建成试运行，建龙钒氮合金一期投产达效，河钢承德钒钛新材料公司、河北钒钛产业技术研究院组建运营。百万千瓦风电基地二期工程加快推进，清洁能源电力装机和发电量分别增长30.6%和13.3%。成功承办第三届河北省旅发大会，建成中国马镇、小滦河湿地公园等六大组团20个精品项目，国家“一号风景大道”成为承德旅游新地标，带动全域旅游上了一个大台阶，全市游客接待量和旅游总收入分别增长20.9%和26.6%。围场获评国家森林旅游示范县。大力改造提升传统产业。圆满完成83万吨粗钢产能年度压减任务。实施亿元以上工业技改项目95个，绿色改造矿山企业58家。新增规上企业43家，工业主营业务收入、利润总额分别增长11.9%和60.2%，均居全省前列。现代农业稳步发展，粮食总产量145万吨，农产品深加工企业达到94家、产值260亿元，产业化经营率达到70.2%。

　　（三）脱贫攻坚取得重大成果。聚焦“两不愁、三保障”，持续打好八措并举“组合拳”，脱贫攻坚向纵深推进。脱贫成效更加明显。全市14.31万贫困群众实现稳定脱贫，贫困发生率下降到2.52%，平泉市高质量脱贫出列，兴隆、滦平、承德县达到贫困县退出标准、完成省级验收。“政银企户保”、“万户阳光”、“一地生四金”等扶贫模式在全国全省推广。脱贫举措更加精准。大力推进产业扶贫，实施产业扶贫项目2162个，新建村级光伏电站301个，贫困村和贫困户实现产业扶贫全覆盖，全省产业扶贫现场会在承德召开。用好国家东西部扶贫协作机遇，京津两市投入帮扶资金15亿元，实施对口帮扶项目116个。加快实施易地扶贫搬迁，完成141个安置小区建设任务，4.78万人喜迁新居。“632”医疗保障救助扶贫模式全面深化，为195万人次增报医疗费用6.02亿元。脱贫基础更加牢固。深入推进“双基”提升工程，新改建农村公路1200公里，实施电网改造项目959个。发展高效节水灌溉10万亩，建成市级以上美丽乡村精品村150个，平泉和滦平、围场、隆化、承德县分获全国全省“四好农村路”示范县（市）称号。

　　（四）生态环境质量持续改善。坚决扛起建设京津冀水源涵养功能区政治责任，大力弘扬塞罕坝精神，污染防治攻坚战取得显著成效。大气质量进一步提升。淘汰取缔燃煤锅炉2695台，实施农村“双代”2844户，整治“散乱污”企业648家，PM2.5平均浓度32微克、下降8.6%，市区优良天数继续保持京津冀前列。水环境质量进一步向好。深入实施水污染防治“三年百项重点工程”，国省考地表水监测断面Ⅲ类及以上水质比例提高5.3%，出境断面水质达标率保持100%。净土保卫战进一步深化。新增营造林65.6万亩、草地治理修复30万亩，水土流失治理654平方公里；修复绿化矿山8平方公里，压减矿权67个，关停取缔矿山30家。生态文明体制机制进一步完善。首部地方立法《承德市水源涵养功能区保护条例》颁布实施，潮河生态补偿机制取得突破性进展，领导干部自然资源资产离任审计和绿色GDP核算试点逐步推开，具有承德特色的生态文明法制和制度体系初步建立。

　　（五）改革创新蹄疾步稳纵深推进。坚持以改革创新引领经济社会发展，发展的动力活力不断增强。创新实施“承德办事一次成”改革。以自我革命的勇气，积极探索“放管服”改革的承德路径，公开权力、再造流程、智慧政务多措并举，全市平均75%的行政权力事项和100%的公共服务事项进入“一次办成”清单。大刀阔斧出台改革举措，中心城区房屋建筑和城市基础设施工程审批时限从210个工作日压缩至24个工作日，企业开办时间从30个工作日压缩至4.5个工作日，营商环境得到改善和优化，新增市场主体2.6万户、其中企业8390户。“双创双服”取得扎实成效。认真落实全省“双创双服”活动，制定出台“1+33+1”系列工作方案，创新实施“走千企帮万家”行动，市县两级全部成立企业发展服务中心，为企业解决问题1246个。减轻企业负担4.5亿元，支持企业融资113亿元，新增年纳税500万元以上企业14家。科技创新取得重大成果。成功争创国家可持续发展议程创新示范区，承德市工业设计创新中心启动运行。新增院士工作站5家，新培育科技型中小企业338家，新认定高新技术企业74家、实现倍增。此外，扎实推进其他重点领域改革，土地流转率达到36.2%，国投集团组建运营，承德演艺集团挂牌成立。

　　（六）协同发展迈出更大步伐。认真落实京津冀协同发展重大国家战略，新时代开放带动战略取得新成效。京津冀协同发展成果丰硕。深化与京津“五合一”精准对接，签约合作项目106个，新增央企和京津产业项目34个、总投资520亿元。与京津合作打造的津冀六沟产业园、怀丰产业园等加快建设，承德应用技术职业学院实现招生办学。交通基础设施一体化全面提速。京沈客专承德至沈阳段开通运行，承德正式跨入高铁时代。承德机场旅客吞吐量达到43万人次，创造出河北民航发展的最快速度。对外开放合作视野不断拓宽。创新扩大开放精准招商机制，聚焦长三角珠三角及“一带一路”沿线地区开展招商合作，深圳市河北承德商会正式成立。成功举办百家名企进承德、深圳招商会、京承和津承扶贫产业协作对接会等系列重大招商活动，累计签约项目167个，引进市外到位资金410亿元。园区聚集力承载力加快提升。全市高新区、开发区新增入园企业197家，固定资产投资和主营业务收入分别增长21%和16%，经济总量占比达到45%。

　　（七）民生保障水平进一步提高。牢固树立和践行以人民为中心的发展思想，尽心竭力为群众解难题、增福祉，民生投入占财政支出的85%，“十大民心工程”20件民心实事全面完成。城镇新增就业5.73万人，新增农村劳动力转移就业5.2万人，高校毕业生就业率达到95.1%，城镇登记失业率3.69%。城乡居民基本医疗保险、养老保险参保率分别达到96%和98.5%，城乡低保年标准分别提高到7200元和3900元。改造棚户区9182套、农村危房2.7万户，超额完成省定任务。解决房地产领域遗留问题项目267个，改造供热供水燃气老旧管网53.5公里，新增集中供热面积580万平方米。新建改扩建学校幼儿园112所，中小学校课后延时服务在全市推开，8.8万名中小学生和家庭从中受益。所有县（市）区全部完成义务教育均衡发展国家验收。承德博物馆、冰上运动中心基本建成，进入布展、验收阶段。启动实施高雅艺术进承德活动，天津交响乐团等多个知名艺术团体为承德人民献上精神文化大餐。全国文明城市创建在全省测评中取得好成绩。法治承德、平安承德建设扎实推进，扫黑除恶专项斗争深入开展，安全生产、文物保护、食品药品安全以及动物疫情防控形势总体平稳，群众安全感继续保持全省第一，社会和谐稳定局面进一步巩固。

　　一年来，我们坚决落实全面从严治党战略部署，大力推进政府自身建设，认真抓好中央和省委巡视反馈意见和国务院大督查反馈问题整改，严格落实中央八项规定及实施细则精神，加大审计监督力度，政府公信力执行力进一步提升。我们大力防范化解金融风险、债务风险和社会风险，周密防控、果断处置，牢牢守住了不发生系统性区域性风险的底线。我们深入学习宣传和贯彻实施《宪法》，着力增强政府依法行政能力，自觉接受人大法律监督与工作监督、政协民主监督以及社会监督、舆论监督，办理人大代表建议153件、政协提案358件，按时办复率均为100%。我们进一步巩固强化军政军民团结，国防动员、优抚安置工作得到新的加强。人口计生、民族宗教、新闻出版广电、外事侨务、人民防空、史志档案、统计调查、气象地震、防灾减灾、对口援疆、邮政通信、妇女儿童、老龄、残疾人等工作都取得了新成绩。

　　各位代表，过去的一年，我们走过了不平凡的历程。面对经济下行的巨大压力，我们保持定力、砥砺向前；面对生态保护的重大责任，我们执着坚定、勇于担当；面对各种风险和挑战，我们提振士气、攻坚克难；面对历史性宝贵机遇，我们鼓足干劲、奋发作为。成绩来之不易。这是习近平新时代中国特色社会主义思想科学引领的结果，是省委、省政府和中共承德市委正确领导的结果，是全市人民撸起袖子干出来的、挥洒汗水拼出来的。在此，我代表市人民政府，向全市380万人民，向人大代表、政协委员，向各民主党派、工商联、无党派人士和人民团体，向驻承人民解放军、武警部队官兵和政法干警，向一直以来关心和支持承德经济社会发展的国内外各界朋友，致以崇高的敬意和衷心的感谢！

　　我们也清醒地看到，当前经济运行稳中有变、变中有忧，我市长期存在的影响和制约发展的深层次矛盾尚未得到根本解决，经济发展依然面临诸多困难、压力和挑战。主要是：经济总量小、基础差、底子薄，资源依赖型的产业结构状况还没有根本改变，兴市立县的财源支撑项目缺乏，新旧动能转换亟待加快；科技投入不足，创新能力不强、活力不够，高层次人才匮乏，实体经济发展质量不高的问题必须高度重视；各类风险防控形势严峻，安全生产、环境保护、政府债务、金融安全、社会稳定等方面的风险不容忽视；深度贫困地区、特困群体脱贫难度大，精准扶贫、乡村振兴、全面小康的任务十分艰巨；民生领域短板和欠账依然较多，群众日益增长的对美好生活需求与优质公共服务供给不足的矛盾突出；一些政府部门和工作人员思想不解放、能力不适应、作风不扎实，营商环境不优问题和消极腐败现象还不同程度存在。对这些问题，我们必须时刻保持强烈的责任感、紧迫感和忧患意识，采取更加有力措施认真加以解决，以新的发展成效回应全市人民的期盼和重托，不断开创新时代生态强市、魅力承德建设新局面。

　　二、2019年工作总体要求

　　今年是新中国成立70周年，是全面建成小康社会、实现第一个百年奋斗目标的关键之年。今年政府工作的总体要求是：坚持以习近平新时代中国特色社会主义思想为指导，全面贯彻党的十九大和十九届二中、三中全会精神，深入落实习近平总书记对河北对承德重要指示，认真落实省委九届八次全会决策部署，统筹推进“五位一体”总体布局，协调推进“四个全面”战略布局，按照市委十四届五次全会“666”总体要求，坚持稳中求进工作总基调，坚持新发展理念，坚持推动高质量发展，坚持“三区一城”发展定位和“12345”思路目标，以供给侧结构性改革为主线，全面深化改革开放，深入落实“六稳”举措，继续打好“三大攻坚战”，统筹推进稳增长、促改革、调结构、惠民生、防风险各项工作，进一步增强人民群众获得感、幸福感、安全感，加快建设新时代生态强市、魅力承德，以优异成绩庆祝中华人民共和国成立70周年。

　　今年经济社会发展的主要预期目标是：生产总值增长6.5%左右，全部财政收入和公共财政预算收入均增长6.5%，规上工业增加值增长4.5%左右，固定资产投资增长6%左右，社会消费品零售总额增长9.5%左右，进出口总值增长5%；城镇登记失业率控制在4.5%以内；城乡居民人均可支配收入分别增长8.5%和10%；居民消费价格涨幅控制在3%左右；万元生产总值能耗及化学需氧量、二氧化硫、氨氮、氮氧化物减排完成省下达的目标任务。上述目标的设定，综合考虑了外部经济环境和各方面支撑条件，与全省经济指标和我市“十三五”规划目标相衔接，体现了“既积极、又务实”的原则，符合中央和省对承德的定位要求，符合我市的发展实际。

　　做好今年经济社会发展各项工作，必须坚持党对经济工作的领导，进一步推动经济社会发展始终沿着正确方向前进。党中央集中统一领导是我们最大的政治优势和体制优势。必须树牢“四个意识”，坚定“四个自信”，做到“两个维护”，坚决贯彻习近平新时代中国特色社会主义思想和习近平总书记对河北对承德的重要指示，全面落实省委省政府和市委各项决策部署，做到政令畅通、执行坚决、贯彻有力、令行禁止，同心协力把承德的事情办好。必须坚持生态优先绿色发展，进一步树牢践行新发展理念。牢记我们这一代人肩负的职责使命，深刻汲取潮河上游滦平段违法建设问题教训，真正把习近平生态文明思想植根头脑、融入血液、落实在行动上，更加自觉坚定地树牢新发展理念，用新发展理念统一思想认识、谋划推动工作、衡量评价发展成果，坚决摒弃粗放落后的发展方式，坚决扛起承德水源涵养、生态支撑的政治之责、发展之责、民生之责，筑牢承德永续发展的根基命脉。必须准确把握稳中求进总基调，进一步牢牢扭住发展第一要务不放松。目前我市正处在转型升级、转换动能的关键时期，我们要用发展解决前进道路上遇到的各种矛盾和问题，保持战略定力和进取精神，既要“稳”得住，更要善于“进”，以思想观念之“进”、改革创新之“进”、环境政策之“进”，培育新产业、激发新动能、打造新优势、创造新业绩。必须牢牢把握历史机遇，进一步坚定高质量发展的信心决心。习近平总书记亲自谋划推动、全省上下全力推进的京津冀协同发展、雄安新区规划建设、冬奥会筹办“三件大事”，件件都是承德发展的重大机遇；省委省政府明确提出支持我市创建国家可持续发展议程创新示范区、生态旅游创新示范区、绿色矿业发展示范区和钒钛产业基地建设，事事都是承德发展的重要载体；承德机场和京沈高铁的开通、生态引力进一步增强、政通人和万事兴的局面业已形成，项项都是承德发展的宝贵支撑。我们一定要抓住难得的历史性窗口期和战略性机遇期，振奋精神、拼搏进取，坚定不移地走出一条具有承德特色的创新发展、绿色发展、高质量发展之路。必须尽心竭力改善民生，进一步满足人民群众对美好生活的向往。承德作为革命老区、民族地区和欠发达地区，教育、医疗、养老、就业、住房等民生领域的短板欠账比较多。我们要善于听民声、察民情、顺民意、解民忧，始终把人民放在心中至高无上的位置，用心用情用力推动就业创业、脱贫攻坚、教育医疗、社会保障等各项工作，让人民群众有更多实实在在的获得感幸福感安全感，充分激发蕴藏在人民群众中的创造伟力，一起拼搏、一起奋斗，共同向着承德更加美好的明天阔步前进。

　　三、2019年重点工作

　　今年全市改革发展稳定各项任务艰巨繁重，必须把思想和行动统一到中央和省各项决策部署上来，按照市委十四届五次全会确定的目标任务，聚焦主要矛盾，把握关键重点，着力做好九个方面工作。

　　（一）坚定不移强化水源涵养生态支撑，坚决当好首都生态“护城河”。深入学习贯彻习近平生态文明思想，牢牢守住底线，不断调高标尺，深入推进“1+4+10”生态环境问题大排查大整治，确保生态环境质量继续保持京津冀前列。以更高的站位整治水环境。严格落实河湖长责任制，持续推进水污染防治“三年百项重点工程”，全面完成乡镇饮用水源地保护区划定，全部取缔禁养区内畜禽养殖，规模化畜禽养殖场和沿河养殖企业全部配建粪便污水处理设施。中心城市和县城污水处理率分别达到95%和90%，开展农村污水治理工程50个。重点镇具备生活污水、生活垃圾处置能力，地表水国省考核断面水质达标率100%。以更高的标尺打赢蓝天保卫战。继续实施减煤、治企、控车、抑尘、增绿五大攻坚，大力开展散煤治理等12个专项行动，加快淘汰35蒸吨及以下燃煤锅炉，中心城区高污染物禁燃区散煤全部取缔，完成11万户农村清洁取暖洁净煤替代，“散乱污”企业实现动态“清零”。中心城区新增集中供热面积160万平方米，PM2.5平均浓度等环境指标完成省下达任务。以更大的力度实施绿化净化行动。继续深入开展国土绿化三年行动，完成营造林65万亩，治理水土流失面积500平方公里。高标准建设国家绿色矿业发展示范区，深入实施矿业减量化、生态环境修复等“六大工程”，修复裸露矿山4平方公里、压减矿权40个、关停取缔矿山30家。完善固体危险废物管理制度，建立土壤污染风险管控和修复名录，严控化肥、农药使用量，全力维护土壤环境安全。以更严的法纪管控生态环境。积极探索领导干部绿色GDP考核体系，完善领导干部自然资源资产离任审计制度，健全生态环境损害责任终身追究制度，探索跨区域用能权、用水权、排污权等生态产品交易。编制实施潮河、滦河生态保护规划，深入实施塞罕坝机械林场及周边地区可持续发展规划，严肃规划刚性。严格生态管控，认真落实新上项目环境准入负面清单，依法严厉打击各种破坏生态环境行为，加快构建具有承德特色的生态文明体制机制，让生态这块承德的金字招牌更加明亮。

　　（二）坚定不移扭住发展第一要务，多措并举推进经济平稳健康发展。坚持把稳增长作为首要任务，深入开展“双创双服”活动，着力激发有效需求，确保经济运行在合理区间。全力以赴抓投资增后劲。启动新一轮重点项目“三年攻坚行动”，重点推进项目建设“3811”工程，全年争列省重点项目30个以上，实施超亿元高质量项目800个，重点推进超10亿元项目100个，谋划储备亿元以上项目1000个。开展重点项目建设和招商引资“提质、增比、进位”擂台赛，落实重点项目“六个一”包联机制，特变电工新能源、建龙钒钛高科装备等220个重点项目开工建设，荣盛新能源、钛能3D打印等一批重大项目投产运营。围绕打造“新财源建设”工程，加大企业技改扩能力度，实施苏垦银河连杆等亿元以上技改升级项目80个。深入推进小升规、规改股、股上市，力争新增年纳税超亿元企业5家，纳税500万元以上企业10家。千方百计扩大消费新需求。适应服务消费增速加快、比重提升新趋势，深入开展“增品种、提品质、创品牌”行动，扩大教育、信息、网络、养老、医疗等消费，加快城市商业综合体和社区体验店建设，增加高品质商品供给。落实好个人所得税专项附加扣除等提高居民收入政策措施，进一步释放内需潜力。大力推进文化旅游融合发展，探索全市旅游景区景点“一卡通”实现路径，促进旅游消费增量提质。深入推进“互联网+流通”行动，科学布局京津冀一体化实体商超、社区和农村便利店相结合的OTO模式，打造一批精品示范店，支持颈复康、山庄集团等企业建立自主电商平台，全市电子商务交易额增长24%以上，农产品上行规模增长25%以上。毫不动摇发展民营经济。民营经济是发展经济、贡献税收和提供就业的重要力量。坚持把民营企业发展作为振兴实体经济的重中之重，全面贯彻落实支持民营企业发展的政策措施，继续深化以“走千企帮万家”为主要内容的民营企业“暖心助力”活动，促进企业提产业层级、提技术水平，增发展活力、增市场竞争力、增经济效益。严厉打击针对民营企业的强揽工程、恶意围标串标等违法行为，全面清理地方政府部门拖欠民营企业账款。充分发挥企业发展服务中心作用，认真落实国家更大规模减税和更明显降费政策，清理和规范各种附加收费，切实降低实体经济各类营商成本，坚决破除各种“卷帘门”“玻璃门”“旋转门”。着力破解民营企业融资难、融资贵等问题，力争民营企业贷款增长11%以上，民营经济占生产总值比重达到70%。民营企业家是社会的宝贵财富。要积极构建亲清新型政商关系，对民营企业家高看厚爱、真心呵护、倾力支持，着力培养一批具有全球视野、创新能力和社会责任感的本土企业家队伍，让他们更充分地发挥聪明才智、放手放胆创新创业。

　　（三）坚定不移推进供给侧结构性改革，加快构建承德特色绿色产业体系。认真贯彻“巩固、增强、提升、畅通”八字方针，结合落实省政府系列三年行动计划，加快发展全市“3+ 3”绿色主导产业和县域“1+2”特色产业，力争绿色产业增加值占GDP比重提高5个百分点以上，高新技术产业增加值增长12%以上。市级层面，做大做强三大优势产业：文化旅游产业，围绕创建国际旅游城市和国家全域旅游示范市，持续提升国家“一号风景大道”建管运营水平和影响力，高水平办好由双桥、双滦、平泉、隆化共同承办的第三届市旅游文体产业发展大会，所有县（市）区按照国家全域旅游示范区创建标准进行打造。加快发展旅游文创产业，推出一批具有承德特色的旅游文创产品，强化旅游整体宣传推介，精心谋划打造一批旅游精品线路。建设普宁文化休闲小镇等新景区新业态40个，金山岭长城晋升国家5A级景区，加快推进“21世纪避暑山庄”国家级文化旅游产业示范园区创建。进一步提升旅游接待能力，启动双桥区游客服务中心建设。借力借势北京冬奥会，大力发展冰雪产业，加快建设崔梨沟国际滑雪小镇等一批冰雪项目，启动建设“世界塞罕坝、中国沃卡提”等一批体育冰雪运动基地，打造中国马镇冰火节、雪地摩托车锦标赛等一批冰雪活动和赛事品牌，推广普及群众性冰雪运动，扩大承德冬季旅游影响力。全市游客接待量、旅游总收入分别增长18%和22%。钒钛新材料及制品产业，围绕建设国家钒钛产业基地，充分发挥钒钛产业技术研究院等科研机构作用，持续加大钒钛新材料及制品研发力度，推进钒钛产业向高端化、绿色化迈进。支持河钢承德钒钛新材料公司做大做强，加快建设“四大钒钛新材料产业园”，努力打造世界级钒钛产业研发和制造中心。双滦钒钛园承钢120万吨钒钛高强冷轧板一期工程投产达效，高纯氧化钒、硫酸氧钒等高端钒制品项目竣工投产；兴隆、营子建龙钒钛特钢园提钒升级项目加快建设，启动实施钒氮合金二期和含钒高强无缝钢管升级工程；谋划推进隆化钛业园3D打印基地、宽城钒钛特色园高纯金属粉沫等重大项目建设，力争钒钛主营业务收入达到420亿元。清洁能源产业，依托丰富的风电、水电、太阳能资源，抓住国家政策机遇，适度有序发展风电、积极发展光伏发电、深度挖潜发展水电、持续用力推进核电，加快打造国家级清洁能源生产应用产业基地。丰宁抽水蓄能电站投资达到80亿元，加快长河核电前期工作，新增清洁能源电力装机100万千瓦、累计发电量超过100亿千瓦时，全市清洁能源电力装机和发电量占比分别达到75%和50%以上。培育壮大三大特色产业：大数据产业，大力发展存储、清洗、分析、应用、研发全产业链条，加快德鸣大数据产业园、力海企业港产业园、中关村承德创新创业产业园、中电承德信息安全产业园等“四大园区”建设，建成与北京大数据直通直连网络，新投入运营企业15家以上，着力构建大数据产业集群。绿色食品及生物健康产业，深入实施“扶龙工程”，壮大农业产业化龙头企业，带动农产品加工业高质量发展。扶持森源、平安高科、宇航人等龙头企业产品向生物保健、功能饮品等更高层级发展，支持颈复康、天原药业、希力药业等企业开发生物技术药物，绿色食品及生物健康产业产值达到280亿元。特色装备制造产业，积极开展工业设计，培育壮大军民融合产业，推动先进制造业和现代服务业深度融合，装备制造业产值达到100亿元以上。围绕打造中国北方智能仪器仪表生产基地，引导龙智达、克罗尼等仪器仪表企业产品向智能化方向发展。加快特色装备制造产业“一核、四组团”布局，大力发展新能源汽车及汽车零部件、发动机生产制造，支持比亚迪新能源汽车、丰宁汽车配件产业园等龙头企业和园区发展。县级层面，突出差异化特色化发展方向，在大力推进全域发展文化旅游产业的同时，各县（市）区立足自身实际，重点发展2个特色产业，着力延链、补链、强链，培育形成产业集群。谋划推动打造一批市县共享园区和重大项目，加快构建与市级互为依托、互为支撑、互为补充的“1+2”产业体系，打造一批县域经济增长极。强化产业推进和落地保障。大力开展“主导产业振兴年”“项目质量建设年”“园区发展提升年”三大活动，对每个产业逐一制定振兴计划和行动方案，实行“一个工作专班、一个发展规划、一支产业基金、一个招商平台、一本任务台账”的“五个一”推进机制，定期调度、专项督查、考核排队，强力推进主导产业做大做强。大力提升园区承载力，推动产业集群发展。启动实施新一轮开发区改革发展三年提升行动，加快推进园区基础设施建设，所有开发区建成区全部达到“九通一平”，全市开发区新增入园企业180家，固定资产投资和税收收入分别增长15%和16%，开发区经济总量占到全市的“半壁江山”。

　　（四）坚定不移抢抓协同发展重大机遇，打造全方位对外开放新格局。坚持立足京津、面向国内、放眼世界，进一步拓宽对外开放的广度深度，主动参与更大范围的资源配置和市场竞争。提高站位推进协同发展。认真学习贯彻习近平总书记在京津冀协同发展座谈会上的重要讲话精神，坚持以承接非首都功能疏解为“牛鼻子”，持续推动协同发展取得新突破新成效。继续大力实施“五合一”精准对接，精心组织系列对接活动，承接产业转移项目80个以上、新落地50个以上。推动北京协同发展规划直接涉及承德的22个重大事项落实，承德应用职业技术学院建成投用。抓住用好国家在北京周边布局一批“微中心”的难得机遇，高标准谋划建设2—3个特色鲜明的“微中心”和产业园，提高承接能力和水平。探索建立园区与京津“共建共享”模式，推动津冀六沟产业园、怀丰产业园等重点园区建设，确保接得住、发展好。加快推进交通一体化，承平高速全面开工建设。积极主动对接雄安新区规划建设需求，支持我市优质产品走进雄安，学习雄安先进理念和模式发展承德。创新机制深化对外开放。坚持“引进来”精准招商，充分发挥承德机场和京沈高铁（承沈段）开通优势，面向粤港澳大湾区、长三角和东北、成渝地区，主动融入“一带一路”，制定精准招商行动计划，出台高水平开放配套措施。大力推行以商招商、委托代理招商，组织开展好百家名企进承德等系列招商活动，全年引进市外资金440亿元以上，实际利用外资2亿美元以上。坚持“走出去”拓展合作，加强与日本柏市、高崎市和韩国庆州市等友好城市的定期互访，不断拓展双方经贸、科技、旅游、文化等领域合作，每个县（市）区至少引进1家以上外资企业。大力支持企业走出去，推动舒适家用品、神栗食品等企业开拓国际市场，外贸出口额达到5亿美元，进出口总值增长5%，新增外贸出口备案企业40家。统筹城乡推进新型城镇化。积极融入以首都为核心的世界级城市群，坚持以城带乡、城乡统筹、园区支撑、产城融合发展，加快推进新型城镇化步伐。进一步做大做强城市经济，深入实施“一带三区”发展战略，扎实推进国土空间规划编制，稳步推进城市产业布局优化，拓展发展空间，统筹推进城市中心商务区建设和楼宇经济、总部经济发展，实施高铁商圈、临空经济产业区等重大基础设施和产业项目40个，切实增强中心城市发展力和辐射带动力。深入开展文明城市创建，围绕创建标准补短板，对照负面清单强弱项，持续推进全国文明城市创建三年行动计划，常态化实行“全民洗城”，再拆除违建100万平方米，为创城成功奠定坚实基础。加快县城扩容提质，提升县域经济发展水平，增强县城综合承载能力。实施一批海绵城市、旧城整修、综合管网等基础设施项目，力争省级园林县城、卫生县城实现全覆盖。加大撤乡并镇力度，培育打造丰宁凤山、围场四合永等一批特色小城镇，推进农村人口有序向城镇转移。推广平泉市经验，稳步推进中心村建设。城镇化率提高1.2个百分点以上。

　　（五）坚定不移精准扶贫精准脱贫，奋力夺取脱贫攻坚决定性胜利。坚持以提升脱贫质量、巩固脱贫成果和提高群众满意度为重点，进一步聚焦丰宁、围场、隆化3个深度贫困县71个深度贫困村，严格落实“三个新增”要求，确保全市5.5万贫困人口稳定脱贫，所有贫困县脱贫摘帽，全市脱贫攻坚取得决定性胜利。打好产业扶贫关键仗。围绕打造“五个百万基地”“一环六带”扶贫产业带和九大特色扶贫产业，大力推广政银企户保、资产收益、微工厂等十大扶贫模式，扶持扶贫龙头企业100家，打造产业扶贫示范村153个、乡村旅游重点村50个，新增村级光伏扶贫电站330个。培育壮大承德礼仪服务、山庄机电工、热河月嫂、非遗就业工坊等劳务品牌，让贫困群众致富有门路、就业不发愁。聚焦特困群众兜好底。全面落实“三保障”各项措施，扎实推进教育扶贫“三提两培”行动计划，深入实施农村基层医疗卫生“雨润工程”，继续加大“632”医疗保障救助等政策兜底力度，坚决防止因贫失学辍学、因病致贫返贫等问题发生。加快推进易地扶贫搬迁，全面完成162个集中安置小区建设任务，确保剩余5000人全部迁入新居，易地扶贫搬迁全面告捷。巩固脱贫成果防返贫。坚持脱贫攻坚和脱贫巩固“两手抓”，对已脱贫的贫困县、贫困村和贫困户，完善后续巩固提升和精准防贫机制，保持扶贫政策的连续性，切实筑牢“边脱贫、边返贫、边增贫”的“防火墙”。对非高标准脱贫户和非贫困低收入户，强化综合扶持政策，采取有力措施增加收入，防止重返贫困人口行列。深入实施深度贫困地区“双基”提升工程，统筹推进农村基础设施和基本公共服务建设，下大力解决非贫困村和贫困村发展不均衡问题。健全扶贫机制聚合力。用足用好东西部扶贫协作机遇，充分发挥中省直、京津唐对口帮扶作用，加快各项帮扶项目落地落实。坚持扶贫与扶志、扶智相结合，制定后续帮扶和巩固提升规划，落实包保责任机制，增强贫困群众内生动力和自我发展能力。深化扶贫领域腐败和作风问题专项治理，严格资金和项目全程监管，让扶贫工作得到群众认可、经得起历史检验。

　　（六）坚定不移深化改革全面创新，不断增强发展动力活力竞争力。始终把改革创新作为发展的动力之源，全面落实中央和省重大改革部署，以优化营商环境和增强市场活力为重点，锐意改革，强化创新。坚持以“承德办事一次成”改革为重点大力优化营商环境。以更大的决心、更实的举措，持续深化“承德办事一次成”改革。突出问题导向，强化市县联动，巩固改革成果，通过再推出一批举措、再优化一批流程、再精简一批事项、再压缩一批时限的“四个一批”专项行动，全市行政权力事项“一次办成”率达到80%以上，力争所有事项实现网上可办，“一次不用跑”事项扩大到40%，审批时限平均再压减20%。“满意承德”城市综合服务平台开通运行，聘请社会监督员加强改革成效监督，真正实现“一网通办、只进一门、只跑一次”。深入落实“双随机、一公开”，完善信用监管，提升监管效能。加快建立营商环境评价体系，坚决治理不作为、慢作为、乱作为现象，将“承德办事一次成”打造成为承德营商环境的靓丽名片。坚持以“可持续发展议程创新示范区”为牵引大力推动全方位创新。“国家可持续发展议程创新示范区”建设是党中央国务院顺应国际潮流、推动区域创新作出的重大决策部署，承德争创告捷来之不易，下一步创建成功需要付出更大努力。要坚持把示范区创建作为推动全方位科技创新的重要平台和抓手，加快推进协同创新、产业创新、企业创新，围绕“五大重点任务”和“四大行动”，年内落地创建项目40个，确保示范区创建开好局、起好步。深入落实“1+9+N”系列科技创新政策，实施高端人才引进“千人计划”，力争在关键领域取得一批重大科技成果、引进推广一批先进适用技术，推动与京津共建技术创新平台15家以上，引进高端人才300人以上，新增科技小巨人企业8家、高新技术企业45家，新认定科技型中小企业270家。坚持以“打造承德特色品牌”为目标大力实施质量强市。牢固树立大质量观，深入开展文化旅游、制造业、食品农产品等质量提升“十大专项行动”，推动标准、质量、品牌、信誉联动提升。主导或参与制订修订国际标准、国家标准、行业标准7项以上。大力弘扬劳模精神和工匠精神，培育更多“热河工匠”。开展市政府质量奖评选，打造10家质量标杆企业，培育市级以上质量管理专家50名，支持平泉、宽城、双滦争创全国质量强市示范县（市）。坚持以“将改革进行到底”的决心勇气大力实施重点领域改革。聚焦重点领域和关键环节，强化问题导向，坚决破除束缚创新创造的思想障碍、制度藩篱和政策壁垒。加快推进国企国资管理改革。坚持政企分开、政资分开和公平竞争，进一步理顺国有资产管理体制，深化行政事业单位国有资产管理，加强经营性国有资产统一监管，实现国投集团实质性运行，促进国控集团资产保值增值。持续深化财税金融改革。全面实施预算绩效管理，深化财政事权和支出责任划分改革，着力提升金融服务实体经济能力，鼓励支持金融机构向乡镇社区布点，兴业银行承德分行开业运营。支持更多企业在多层次资本市场挂牌上市，力争新增上市企业10家。加快实施社会事业改革。深入推进教育领域综合改革，大力发展公办幼儿园，支持建设民办普惠园，促进城乡义务教育一体化优质发展；积极适应高考制度改革带来的变化和机遇，加快提升普通高中教育教学质量，力争用2—3年时间打造5所以上品牌学校；深入推进职业教育产教融合、校企合作办学模式，在服务特色主导产业、促进就业创业方面发挥重要作用。努力打造高等教育特色品牌，发挥驻承省属和市属高校学科优势，更好的服务承德经济社会发展。深入抓好“四医联动”改革，深化基本医疗保险支付方式改革，把更多救命救急的好药纳入医保。完善国有文化资产管理体制，推进全民健身和全民健康深入融合，积极创建全国全民运动健身示范市（县）。

　　（七）坚定不移实施乡村振兴战略，扎实推进农业农村现代化。牢固树立农业农村优先发展理念，深入落实乡村振兴战略总体规划及五个配套方案，全力做好新时代“三农”工作。加快发展现代农业。深入推进农业供给侧结构性改革“三年行动计划”，进一步培育壮大质量农业、科技农业、绿色农业和品牌农业。全面落实粮食安全责任制，合理调整“粮经饲”结构，再压减籽粒玉米种植30万亩。大力发展设施农业，新增中药材种植6.3万亩。鼓励经营主体开展“三品一标”认证。加快推进农业产业化，推动“双30强”龙头企业做优做强，实施千万元以上产业项目150个，农业产业化经营率达到71.2%。大力推进农村一二三产业融合发展，加快培育休闲农业、乡村旅游等一批新产业新业态，着力打造一批田园综合体，新增市级以上现代农业精品园区6个。全力打造美丽乡村。扎实推进农村人居环境整治“三年行动计划”，大力创建“乡村振兴示范区”，打造市旅发大会沿线美丽乡村30个、精品片区4个，完成所有贫困村环境整治任务。全面推行城乡一体化垃圾收集处理，双桥、双滦、营子和承德高新区实现全覆盖，其他8个县（市）行政村生活垃圾收集处理率达到90%；改造农村无害化厕所22万座，对60个重点邻河村实施农村污水处理改造，实现村庄环境干净、整洁、有序。实施乡村文化特色打造工程，加强历史文化名村、民族民俗、传统村落、历史建筑保护开发，鼓励支持编修镇村史志，保持文化遗产的完整性和延续性，留住更多的乡土记忆，彰显更浓的承德风情。激发农村发展活力。深入宣传贯彻新修订的农村土地承包法，推进承包地“三权分置”改革，大力培育农业新型经营主体，促进适度规模经营，新发展家庭农场200家，评定市级示范家庭农场50家。全面开展农村各类资源资产确权，规范农村产权交易行为，促进农村经济健康发展。推进“农业产业化联合体”建设，深入实施合作社规范提升工程，新认定市级以上农村股份制合作经济组织100家。稳步推进农垦改革，加快垦区集团化、牧场企业化进程。大力培育新型职业农民，加强农民实习实训基地建设，培养一批农业职业经理人和新乡贤。大力弘扬社会主义核心价值观，倡导移风易俗，促进形成文明家风、良好乡风、淳朴民风，培树一批文明家庭和文明个人，县级文明村占比达到60%以上。

　　（八）坚定不移防范化解重大风险，全力保持社会大局稳定。坚持底线思维，强化防范风险的先手，准备化解风险的硬招，加强各类风险影响和危害的宣传，牢牢把握风险防控主动权。有效防控金融风险。继续深化融资性担保机构、小额贷款公司、互联网金融等重点领域监管，建立健全监测预警和早期干预机制，持续做好防范、打击和处置非法集资工作，进一步优化金融发展环境，坚决守住不发生系统性区域性金融风险的底线。加快建设促进房地产市场平稳健康发展的长效机制，合理引导住房消费预期，整顿规范市场秩序，促进房地产市场平稳健康发展。严格防控债务风险。持续强化债务防控化解制度刚性约束，将政府债务收支全部纳入预算管理，适度控制政府投资规模和标准，稳妥化解存量债务。牢固树立过“紧日子”思想，厉行节约，继续压减政府一般性支出和“三公”经费，提高财政资金使用效益，积极探索吸收社会资本及民营资本参与基础设施建设，相关县区加快退出风险预警、提示名单。切实防控安全风险。严格落实安全生产责任制，聚焦矿山、危险化学品、道路交通、消防安全、森林草原防火等重点行业领域，深入开展安全生产“1+6”专项排查整治行动，建立专业化常态化问题隐患排查整治机制，推动安全生产形势持续稳定向好。突出防控社会风险。全面深化法治承德、平安承德建设，健全完善预防化解社会矛盾机制和社会治安防控体系，深入推进扫黑除恶专项斗争，坚决筑牢首都政治和安全稳定“护城河”。

　　（九）坚定不移保障和改善民生，持续办好人民群众关心关注的实事。时刻把群众的安危冷暖放在心上，从人民群众关心的事情做起，本着既量力而行又尽力而为的原则，集中力量实施十项民心工程，再办20件民心实事。一是就业和社会保障工程。①全市城镇新增就业4.56万人以上，失业人员再就业1.5万人，困难人员实现再就业7000人；城镇登记失业率控制在4.5%以内，高校毕业生就业率保持在90%以上。②大规模开展职业技能培训，全年培训各类技能人才1.32万人次，开展各类创业就业培训4万人次以上。③城乡居民养老保险参保率保持在95%以上，各类保险待遇发放率均达到100%。④改扩建居家养老服务中心16家，全面提升服务能力和水平。二是教育提升工程。⑤新建、改扩建幼儿园60所，认定普惠性幼儿园20所；新建扩建义务教育中小学29所，其中乡村学校20所，高新区冯营子初中、上板城小学、闫营子小学和西大庙小学等4所学校和双桥区世纪城小学竣工投用，双桥区老西营小学、现代城小学、双峰寺镇中学等3所学校和市特教学校完成主体工程。⑥积极推动老年教育与现有教育资源共享，建设老年学校60所。三是医疗健康雨润工程。⑦继续完善基层医疗帮扶机制，市县再选派500名医师，精准帮扶100个基层医疗机构，培养引进基层卫生人才1000人，补充更新医疗设备350台件。把31种抗癌新药列入门诊特殊病用药报销范围。四是济困扶残工程。⑧城乡低保年标准分别提高600元和400元，达到7800元和4300元。为3000名贫困残疾人提供基本康复服务，为2000名贫困残疾人适配基本型辅助器具，为500名贫困残疾人提供托养服务补贴。五是便民和食品安全工程。⑨食品及食用农产品集中交易市场整治提升率达到70%以上，创建省级校园食品安全标准食堂6家、市级50家，新建生鲜超市、农贸市场20个。六是百姓安居工程。⑩新开工棚户区改造2152套，农村危房改造18385户。？高标准改造老旧小区200个，其中中心城区完成中兴路组团、双柳小区、口腔医院家属楼、滦电生活区等30个老旧小区改造，完成100个老旧小区物业服务管理提升，完成广电路、万华B区、财苑小区等11处危险边坡护坝抢险加固。？改造供水、供热、燃气、排水老旧管网168公里。七是人居环境改善工程。？持续美化中心城区，新建武烈河两岸坝体文化墙300延长米，实施广仁岭大街等园林绿化工程23项，其中立体鲜花造景50处，对迎宾路、武烈路、滨河大街等20条街路进行亮化，重点区域实施节日装点，全面提升城市美化亮化水平。？新建改建旅游厕所180座、城区公厕50座。八是道路畅通工程。？普通干线公路建成通车100公里，新改建农村公路1000公里，实施公路生命安全防护工程315公里。？行政村主街道硬化率达到100%，贫困村所辖自然村道路硬化率达到90%以上。？中心城区实施城市道路建设项目24项，年内完成碧峰门路、冯营子次干路、石洞子沟小学过街天桥、承朝高速双峰寺出口平改立等11条道路建设，其中承下线一期完成冀东花园至汽车东站1条主干道和冀东花园北路、汽车东站北路、乾正家园北路3条连接线建设；新开工建设凤凰山大桥拓宽改造、红石砬沟口平改立、小老虎沟隧道等12个城市道路工程项目。？调整优化市区至高铁站的67路、68路等公交线路6条，完成中心城区31个停车场智能化改造，新建立体车库2个，新增停车位1000个。九是文体惠民工程。？组织各类惠民演出活动1000场次，高雅艺术进承德演出3场以上。开展大型群众性体育赛事和活动22项，为200个行政村配建体育健身设施。十是平安承德工程。？住宅小区实施智能化改造10个，创建群众满意社区（农村）警务室100个，创建无矛盾纠纷激化的行政村1000个，让老百姓平平安安过日子，生活得更踏实。

　　各位代表，让人民过上幸福美好生活，是我们一切工作的出发点和落脚点。我们一定要以造福人民为最大政绩，坚决兑现民生承诺，让广大人民群众的获得感幸福感安全感更加完善、更有保障、更可持续。

　　各位代表，新时代要有新气象，更要有新担当新作为。我们将不忘初心、牢记使命，坚持和加强党的全面领导，不断强化宗旨意识，忠诚履职、诚实守信、勤勉尽责，努力建设职能科学、结构优化、廉洁高效、人民满意的服务型政府。我们一定要旗帜鲜明讲政治，坚决维护习近平总书记党中央的核心、全党的核心地位，坚决维护党中央权威和集中统一领导，自觉在政治立场、政治方向、政治原则、政治道路上同以习近平同志为核心的党中央保持高度一致，严守政治纪律政治规矩，坚决履行管党治党政治责任，深入落实意识形态工作责任制，确保党中央国务院、省委省政府和市委的重大决策部署落地见效，在履行首都政治“护城河”职责中体现新担当。我们一定要坚持依宪施政依法行政，弘扬宪法精神，树立宪法权威，忠实履行宪法和法律赋予的职责，深入推进法治政府建设，全面提高运用法治思维和法治方式推进工作的能力和水平，自觉接受人大法律监督、工作监督和政协民主监督，自觉接受人民群众监督和社会舆论监督，在为民用权、为民服务、为民谋利中实现新作为。我们一定要实干担当掷地有声抓落实，把今年作为“全面落实年”，坚持对标看齐、问题导向、比学赶超，构建上下贯通、层层落实的责任链条，推动生态保护、项目建设、产业培育、环境优化等重点工作落地见效，努力提高政府执行力公信力。大力弘扬塞罕坝精神，敢于挑最重的担子，善于啃最硬的骨头，以功成不必在我、功成必定有我的境界，以不畏困难、拼搏奋进的决心和勇气，以创先争优、干则必成的姿态和精神，一步一个脚印推动落实，以新担当新作为努力创造出无愧于党、无愧于人民的新业绩。我们一定要持之以恒正风肃纪，坚持深入实际、深入基层调查研究，倾听民声、了解民情、汇聚民智，尽职尽责干好每一天、用情用力做好每件事。严格执行中央八项规定及实施细则精神，驰而不息反“四风”、树新风，坚决反对和克服形式主义官僚主义，深入推进国企国资、工程招投标等专项治理，加大审计监督力度，以永远在路上的坚韧和执着全面加强反腐倡廉建设，努力造就一支忠诚干净担当的高素质公务员队伍，树立起新时代好干部新形象。

　　各位代表，推动实现承德高质量发展的历史重任鞭策着我们，承德380万人民的殷切期盼激励着我们。使命在肩，初心如磐。让我们更加紧密地团结在以习近平同志为核心的党中央周围，在中共承德市委的坚强领导下，锐意进取，埋头苦干，以永不懈怠的精神状态和一往无前的奋斗姿态，奋力开创新时代全面建设生态强市、魅力承德新局面，以优异成绩庆祝中华人民共和国成立70周年！
